# Network-based mapping and neurotransmitter architecture of brain gray matter correlates of extraversion

**DOI:** 10.3389/fnsys.2025.1640639

**Published:** 2025-10-03

**Authors:** Hai-Hua Sun, Hu-Cheng Yang, Xiao-Yi Liu, Feng-Mei Zhang, Shu Wang, Zhen-Yu Dai, Si-Yu Gu, Ping-Lei Pan

**Affiliations:** ^1^Department of Geriatrics, Affiliated Hospital 6 of Nantong University, Yancheng Third People’s Hospital, Yancheng, China; ^2^Department of Radiology, Affiliated Hospital 6 of Nantong University, Yancheng Third People’s Hospital, Yancheng, China; ^3^Department of Radiology, Binhai Maternal and Child Health Hospital, Yancheng, China; ^4^Department of Neurology, Affiliated Hospital 6 of Nantong University, Yancheng Third People’s Hospital, Yancheng, China

**Keywords:** extraversion, gray matter, functional connectivity network mapping, default mode network, frontoparietal network, neurotransmitter

## Abstract

**Objective:**

To identify common functional brain networks underlying heterogeneous gray matter (GM) correlates of extraversion and to characterize the neurotransmitter receptor and transporter architecture associated with these networks.

**Methods:**

A systematic literature search identified 13 voxel-based morphometry (VBM) studies reporting GM correlates of extraversion in healthy individuals (*N* = 1,478). Functional connectivity network mapping (FCNM) approach using normative resting-state functional MRI data from the Human Connectome Project (HCP, *N* = 1,093) mapped divergent GM correlates extraversion onto common networks. Robustness was assessed via replication using an independent Southwest University Adult Lifespan Dataset (SALD, *N* = 329) and sensitivity analyses varying seed radii. Spatial relationships between the identified brain networks and the distribution of major neurotransmitter receptors/transporters were subsequently characterized using the JuSpace toolbox.

**Results:**

FCNM analysis revealed that reported GM correlates of extraversion converge onto specific functional networks. Spatial overlap analysis showed the highest association with the frontoparietal network (FPN) (37.32%) and the default mode network (DMN) (32.99%). Furthermore, JuSpace analysis indicated that these extraversion-linked networks exhibited significant positive spatial correlations with 5-hydroxytryptamine receptor 2A (5HT2a; *p* = 0.021, *r* = 0.215), cannabinoid receptor type-1 (CB1; *p* = 0.005, *r* = 0.392), and metabotropic glutamate receptor 5 (mGluR5; *p* = 0.01, *r* = 0.330), and negative correlations with the norepinephrine transporter (NAT; *p* = 0.018, *r* = −0.221) and serotonin transporter (SERT; *p* = 0.023, *r* = −0.201).

**Conclusion:**

Despite regional heterogeneity in prior VBM studies, structural GM correlates of extraversion consistently map onto the DMN and FPN. This network-based approach reconciles previous inconsistencies and highlights the importance of these large-scale networks as a plausible functional substrate underlying structural variations associated with extraversion. These findings advance a systems-level understanding of the neural basis of this core personality dimension and suggest a distinct neurochemical architecture within these networks.

## Introduction

Understanding individual differences in personality is crucial, as these fundamental traits consistently predict a wide array of consequential life outcomes, ranging from social functioning and subjective well-being to occupational success and even physical health (Roberts et al., 2007; Wilmot et al., 2019). Extraversion, a core dimension within the widely accepted Five-Factor Model (McCabe and Fleeson, 2012), describes the tendency towards sociability, assertiveness, positive emotionality, and sensation-seeking. Given its relative stability across the lifespan and significant heritability (Ebstein, 2006; Van den Berg et al., 2016), researchers have increasingly sought to uncover the neurobiological substrates underlying this fundamental trait.

Structural neuroimaging, particularly voxel-based morphometry (VBM), has been extensively used to identify potential gray matter (GM) volume correlates of extraversion. However, the results have been notably inconsistent (DeYoung et al., 2010; Li et al., 2019; Lu et al., 2014; Grodin and White, 2015; Kapogiannis et al., 2013; Zou et al., 2018; Andari et al., 2014; Cremers et al., 2011; Forsman et al., 2012; Nostro et al., 2017; Coutinho et al., 2013; Omura et al., 2005). While some studies have reported positive associations between extraversion and GM volume in brain regions like the orbitofrontal cortex (OFC) and amygdala (Cremers et al., 2011; Omura et al., 2005), others have found negative associations or null results in these same areas (Chen and Canli, 2022). This heterogeneity likely stems from methodological variations, differences in sample characteristics, psychometric assessment tools, and the inherent limitations of small sample sizes common in neuroimaging research (Chen and Canli, 2022; Lai et al., 2019). Consequently, a clear neuroanatomical map of extraversion based solely on regional VBM findings remains elusive (Chen and Canli, 2022).

The challenge of synthesizing heterogeneous localization data is not unique to personality neuroscience; it is common across studies linking brain structure alterations to symptoms or traits in neurological and psychiatric disorders (Menon, 2011). A promising approach to overcome this limitation involves leveraging the brain’s network organization, positing that structurally diverse locations associated with a specific function or symptom often belong to a common, functionally interconnected brain network (Fox, 2018). Specifically, functional connectivity network mapping (FCNM) integrates reported coordinates of structural or functional alterations with large-scale human brain connectome data, typically derived from resting-state functional MRI (rs-fMRI) datasets (Zhang et al., 2024; Yang et al., 2025). By projecting disparate neuroimaging findings onto these normative functional connectivity (FC) maps, FCNM can reveal whether seemingly scattered locations converge onto specific large-scale systems. This network localization approach has been successfully applied to map various neurological symptoms and psychiatric conditions, including schizophrenia (Cheng et al., 2025) and suicide-related brain alterations (Zhang et al., 2024), providing coherent neurobiological insights despite initial heterogeneity in regional findings.

However, identifying the relevant large-scale networks is an essential but intermediate step. A deeper neurobiological understanding requires probing the molecular mechanisms, particularly neurotransmission, that might govern the function and susceptibility of these networks in relation to personality like extraversion. The operational dynamics within brain systems are intrinsically modulated by neurotransmitter activity. Key neurotransmitter systems, including serotonergic, dopaminergic, noradrenergic, glutamatergic, and endocannabinoid systems, have been extensively implicated in modulating mood, arousal, reward sensitivity, and social interaction—all core facets of extraversion (Canli, 2004; Terbeck et al., 2015; Shao and Zhu, 2020; Niswender and Conn, 2010; Gillihan et al., 2007). Therefore, examining the spatial relationship between extraversion-linked brain networks and the neurochemical landscape offers a critical opportunity to bridge macro-level network organization with micro-level molecular underpinnings.

Therefore, in this exploratory study, we applied the FCNM approach to synthesize the existing VBM literature on extraversion into common functional networks and subsequently investigated the neurotransmitter architecture of the identified networks. By identifying the large-scale networks most robustly associated with extraversion-related GM variations and their associated neurochemical profiles, this study aimed to provide a more coherent, multi-level understanding of its neural architecture. Such an approach holds the potential to reconcile previous inconsistencies in regional GM findings and guide future investigations into the neurobiological underpinnings of this core personality dimension and its implications for behavior and well-being.

## Methods

### Literature search and selection

Following the PRISMA guidelines, we conducted a comprehensive and systematic search of the PubMed, Embase and Web of Science databases to identify studies on GM correlates of extraversion, published prior to February 3, 2025. The search included keywords such as “voxel-based morphometry” OR “VBM” OR “gray matter” OR “grey matter” AND “extraversion.” We also conducted a manual examination of the bibliographies from pertinent review articles and meta-analyses to capture any studies that may have been missed through the primary search strategy. A flow diagram of the study selection process is shown in Supplementary Figure S1. All studies were included according to the following criteria: (a) published in an English-language peer-reviewed journal as an original article; (b) healthy subjects were examined; (c) used extraversion as the research variable; (d) used voxel-based analysis; (e) reported whole-brain results and regional GM correlates of extraversion; (f) studies that report results in Talairach or Montreal Neurological Institute (MNI) space.

Exclusion criteria were as follows: (a) were non-original studies (e.g., review, meta-analysis, meeting abstract); (b) no coordinate system reported; (c) only reported region of interest (ROI) results; (d) all reported coordinates outside GM mask.

### Discovery and validation datasets

The Human Connectome Project (HCP) 1,200 Subjects Release (S1200) served as the discovery dataset in this study, while the Southwest University Adult Lifespan Dataset (SALD) provided an independent dataset for cross-scanner validation. The HCP dataset consisted of imaging data from 1,093 healthy adults [594 females; mean (SD) age 28.78 (3.69)], with ages ranging from 22 to 37 years. The SALD included 329 healthy adults [207 females; mean age 37.81 (13.79) years]. The exclusion criteria included MRI contraindications, current psychiatric or neurological disorders, use of psychiatric drugs within the past 3 months, pregnancy, or a history of head trauma. The demographic characteristics of the discovery and validation datasets are detailed in Supplementary Table S1.

### Rs-fMRI data acquisition and preprocessing

Rs-fMRI data from the HCP were acquired with a 3 T Siemens Trio MRI scanner, and SALD data with a 3 T Siemens MAGNETOM Trio scanner. Both datasets provided high-resolution images suitable for detailed analysis. The specific fMRI parameters of the 2 datasets are provided in Supplementary Table S2. Participants with images of insufficient quality, including those affected by artifacts or lacking complete brain coverage, were excluded from the analysis.

Resting-state fMRI data were preprocessed using SPM12 software^1^ and DPABI.^2^ The first 10 volumes of each participant’s functional scan were discarded to allow for T1 equilibration effects and participant adaptation to the scanner environment. The remaining volumes were corrected for differences in slice acquisition times using slice-timing correction procedures. Then, realignment was performed to correct the motion between time points. Head motion parameters were calculated by estimating the translation in each direction and the angular rotation on each axis for each volume. We confirmed that head motion parameters for all participants were acceptable, not exceeding 2 mm in maximum translation or 2° in maximum rotation at any point during the scan. We also calculated framewise displacement, which indexes the volume-to-volume changes in head position. Nuisance regression involved removing linear drift, the 24 Friston motion parameters, spike regressors (for volumes with FD > 0.5 mm), global signal, and mean white matter and cerebrospinal fluid signals from the preprocessed time series. Because global signal regression can enhance the detection of system-specific correlations and improve the correspondence to anatomical connectivity, we included this step in the preprocessing of resting-state fMRI data. Then, the datasets were bandpass filtered using a frequency range of 0.01 to 0.1 Hz. In the normalization step, individual structural images were first co-registered with the mean functional images; the transformed structural images were then segmented and normalized to MNI space using a high-level nonlinear warping algorithm, i.e., the diffeomorphic anatomical registration through the exponentiated Lie algebra technique (Ashburner, 2007). Subsequently, each filtered functional volume was spatially normalized to MNI space using the deformation parameters obtained in the previous step and resampled to a 3-mm isotropic voxel grid. Finally, all data were spatially smoothed with a Gaussian kernel of 6 × 6 × 6 mm^3^ full width at half maximum.

### FCNM analysis

We utilized the FCNM approach to map divergent GM correlates extraversion to common dysfunctional networks. First, spheres with a 4-mm radius were centered at each coordinate of a contrast, and these spheres were combined to create a contrast-specific seed mask (henceforth referred to as the contrast seed). Next, seed-to-whole brain FC maps were generated for each participant based on the preprocessed rs-fMRI data provided by the HCP. This process included computing Pearson’s correlation coefficients between the seed’s time series and all brain voxels, followed by a Fisher’s Z-transformation to enhance normal distribution. Third, the FC maps from 1,093 subjects were analyzed using a voxel-wise one-sample *t*-test to pinpoint brain regions functionally linked to each seed. We focused solely on positive FC, as the interpretation of negative connectivity remains controversial (Murphy et al., 2009; Murphy and Fox, 2017). Fourth, group-level *t*-maps were thresholded (*p* < 0.05, voxel-level false discovery rate corrected) and binarized. These resulting binary maps, reflecting significant GM correlates of extraversion, were merged to construct a network probability map. This map was then thresholded at 50% overlap (i.e., regions connected to at least 50% of the contrast seeds), a threshold validated in previous FCNM studies (Cheng et al., 2025; Peng et al., 2022), to delineate the networks of GM correlates of extraversion.

### Association with canonical brain networks

For ease of interpretability, we examined the spatial relationships extraversion brain dysfunctional networks and 8 well-recognized canonical brain networks. The 7 cortical networks were defined as the visual network, somatomotor network, dorsal attention network, ventral attention network, limbic network, frontoparietal network (FPN), and default mode network (DMN) according to the Yeo et al. study (Yeo et al., 2011). The Human Brainnetome Atlas (Fan et al., 2016) was utilized to delineate the subcortical network. We quantified their spatial relationships by calculating the ratio of overlapping voxels between each extraversion dysfunctional network and the respective canonical network to the total number of voxels within those canonical networks. The identified functional network was regarded as significantly involving the corresponding canonical network if their overlap proportion was 20% or more.

### Validation analyses

We conducted several validation analyses to test the robustness of our findings. Frist, We replicated the analyses using an independent validation dataset (the cross-scanner SALD) to assess the robustness of the findings across different datasets. Subsequently, we repeated the FCNM analysis employing spheres with 1-mm and 7-mm radii to evaluate the influence of seed size.

### Correlating networks with neurochemical atlases

To elucidate the relationship between the extraversion mapping network and chemoarchitectonic organization, we conducted spatial correlation analysis through 30 PET-derived maps of neurotransmitter receptors/transporters available in JuSpace version 1.5^3^ (Dukart et al., 2021). The statistical significance of the correlation was determined using a permutation test of 5,000 iterations across all analyses, corrected by false discovery rate (FDR) with *p* < 0.05.

## Results

### Included studies and sample characteristics

Initially, 243 candidate articles were identified and underwent a rigorous screening process. Ultimately, 13 VBM studies including 1,478 participants (768 females, 710 males) were included for the subsequent FCNM analysis. The sample size ranged from 30 to 364 participants, with gender ratio from all-male to balanced or female-skewed distributions, and mean age spanning young adults around 20 years old to older adults aged 72, with a weighted average age across all participants of approximately 27.72 years. A variety of psychometric scales were employed to assess extraversion, with the Revised NEO Personality Inventory (NEO-PI-R) and NEO Five Factor Inventory (NEO-FFI) being frequently utilized (McCrae and Costa, 1992). Regarding imaging parameters, studies predominantly used 3.0 T (*N* = 9) and 1.5 T (*N* = 4) scanners. In data analysis, age and gender were common nuisance covariates, with some studies also controlling for total GM/intracranial volume. Most studies adopted multiple comparison correction methods for their VBM statistical analyses (*N* = 11). Detailed sample and imaging characteristics of the included studies are summarized in Table 1.

**Table 1:** Sample and imaging characteristics of the studies included extraversion analysis.

Included study	Sample size	Ratio F/M	Mean age (SD)	Scales	Scanner/FWHM (mm)	Nuisance covariate	Statistical analysis/*p*-value corr
Andari et al. (2014)	30	13/17	23.57 (3.70)	NEO-PI-R	1.5 T/8	Gender, age, TGMV	*p* < 0.05, nonstationary corr.
Coutinho et al. (2013)	52	29/23	25.0 (5.1)	NEO-FFI	1.5 T/10	Gender, age, TIV	GLM/*p* < 0.05, Monte Carlo corr
Cremers et al. (2011)	65	42/23	40.5 (9.7)	NEO-FFI	3.0 T/8	Age, scan center, TGMV	*p* < 0.05, FWE corr and *p* < 0.001, uncorr
DeYoung et al. (2010)	116	58/58	22.9 (5.5)	NEO-PI-R	3.0 T/8	Gender, age, TGMV	GLM/*p* < 0.05, cluster-size corr (Monte Carlo simulation)
Forsman et al. (2012)	32	0/32	33.2 (7.8)	16PF	1.5 T /12	Age	GLM/*p* < 0.05, FDR, corr
Grodin and White (2015)	83	46/37	24.9 (7.7)	MPQ-BF	3.0 T/12	Gender, age, alternate extraversion traits	*p* < 0.05, FDR, corr & *p* < 0.001, uncorr
Kapogiannis et al. (2013)	87	42/45	72 (7.7)	NEO-PI-R	1.5 T /12	Gender, age, TIV, years of education	GLM/*p* < 0.05, FWE corr
Li et al. (2019)	337	189/148	20.0 (1.3)	NEO-PI-R	3.0 T/10	Gender, age, other four traits, TGMV	*p* < 0.05, FWE corr
Lu et al. (2014)	71	37/34	22.35 (1.5)	EPQ-RSC	3.0 T/8	Gender, age, TIV	GLM/*p* < 0.05, AlphaSim corr
Omura et al. (2005)	41	22/19	23.8 (5.4)	NEO-PI-R	3.0 T/12	Gender, age	GLM/*p* < 0.001, uncorr
Nostro et al. (2017)	364	182/182	29.1 (3.45)	NEO-FFI	3.0 T/8	Gender, age, TIV	GLM/*p* < 0.05, FWE corr
Hsu et al. (2018)	100	58/42	22.0 (2.33)	EPQ-RSC	3.0 T/−	Gender, age, TIV	GLM/*p* < 0.05, FDR corr
Zou et al. (2018)	100	50/50	21.91(2.29)	EPQ-RSC	3.0 T/6	Gender, age	GLM/*p* < 0.05, FWE corr

16PF, Sixteen Personality Factor Questionnaire; corr, correction; EPQ-RSC, Eysenck Personality Questionnaire-Revised Short Scale for Chinese; F, female; FDR, false discovery rate; FWE, family-wise error; FWHM, full width at half maximum; GLM, general linear model; GRF, Gaussian random field; M, male; MPQ-BF, Multidimensional Personality Questionnaire Brief Form; NEO-FFI, NEO Five Factor Inventory; NEO-PI-R, Revised NEO Personality Inventory; TGMV, total gray matter volume; TIV, total intracranial volumes; uncorr, uncorrection.

### Convergent FC associated with GM correlates of extraversion

**Figure 1:**
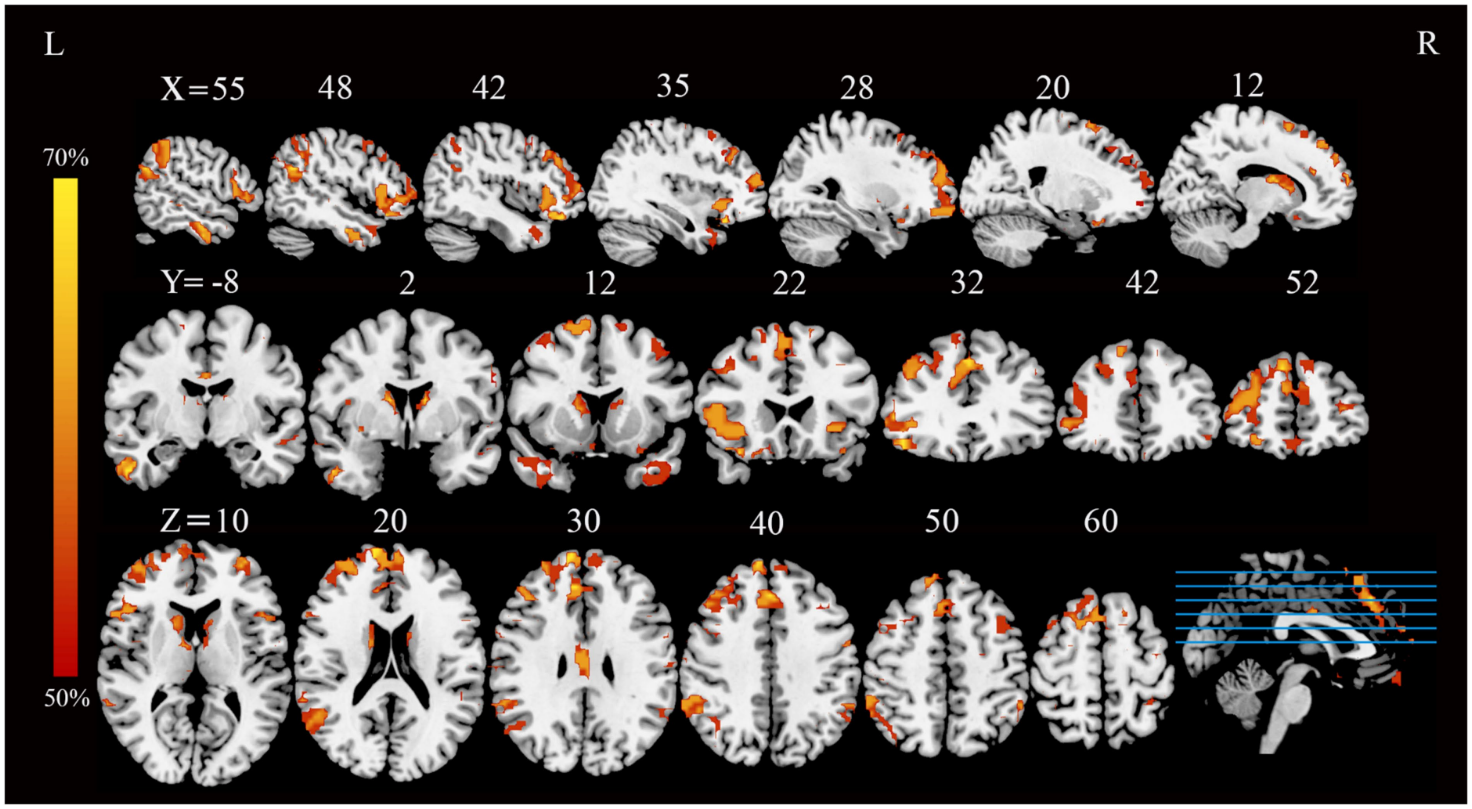
FC overlap maps associated with extraversion (based on a 4-mm radius sphere). Functional brain networks are presented as FC probability maps thresholded at 50%, highlighting brain regions demonstrating FC with more than 50% of the contrast seeds (derived from extraversion-related gray matter coordinates). FC, functional connectivity; L, left; R, right.

Application of the FCNM approach, integrating coordinates of reported GM correlates of extraversion from the 13 VBM studies with the normative HCP connectome, revealed convergent FC associated with extraversion. These FC patterns involved widely distributed brain regions, primarily including the bilateral medial prefrontal cortex (mPFC), right angular gyrus, bilateral dorsolateral prefrontal cortex (DLPFC), bilateral frontoinsular cortex (FIC) and bilateral posterior parietal cortex (PPC) (*p* < 0.05, FDR corrected) (Figure 1).

### Association with canonical brain networks

**Figure 2:**
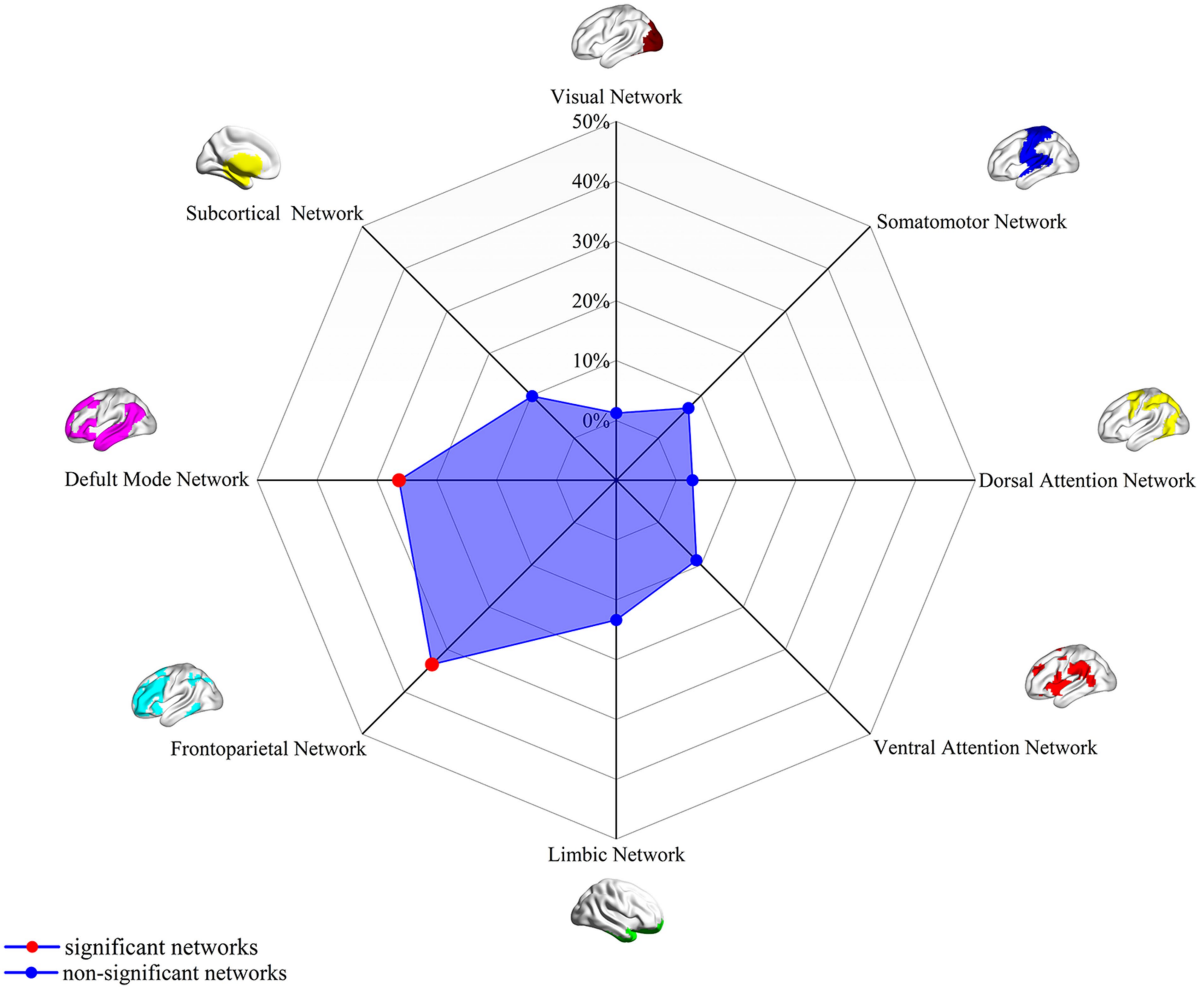
Association of extraversion-related FC overlap maps (4-mm radius sphere) with canonical brain networks. Polar plots illustrate the proportion of overlapping voxels between the extraversion-associated FC map (derived as in Figure 1) and each canonical brain network, relative to the total number of voxels within the respective canonical network. Red dots represent networks with significant overlap (defined as ≥ 20% overlap with the canonical networks), while blue dots represent networks with non-significant overlap (<15% overlap). FC, functional connectivity.

Analysis of the spatial overlap between the extraversion-related aberrant FC and established canonical brain networks indicated preferential involvement of specific systems. The network showed the highest overlap with the FPN, largely corresponding to the bilateral DLPFC, bilateral FIC, and bilateral PPC (overlap proportion: 37.32%). Significant overlap was also observed with the DMN, primarily involving the bilateral mPFC and right angular gyrus (overlap proportion: 32.99%) (Figure 2). Overlap proportions with the remaining canonical networks (visual, somatomotor, dorsal attention, ventral attention, and limbic, subcortical networks) were all below 20%.

### Robustness analyses

We conducted several validation analyses to test the robustness of our findings. Our initial analysis revealed that extraversion-related functional networks derived from the validation SALD dataset closely resembled those from the discovery HCP dataset, with minor variations likely stemming from differing sample sizes (1,093 vs. 329) (Supplementary Figures S2 and S3). Subsequently, FCNM analyses repeated using seed spheres with 1-mm and 7-mm radii yielded topographically similar network patterns to those obtained using the standard 4-mm radius sphere (Supplementary Figures S4 and S5, respectively). Furthermore, the pattern of canonical network involvement remained consistent when replicating the FCNM procedure with spheres of 1-mm and 7-mm radii (Supplementary Figures S6 and S7, respectively).

### Neurotransmitter architecture of extraversion-linked networks

**Figure 3:**
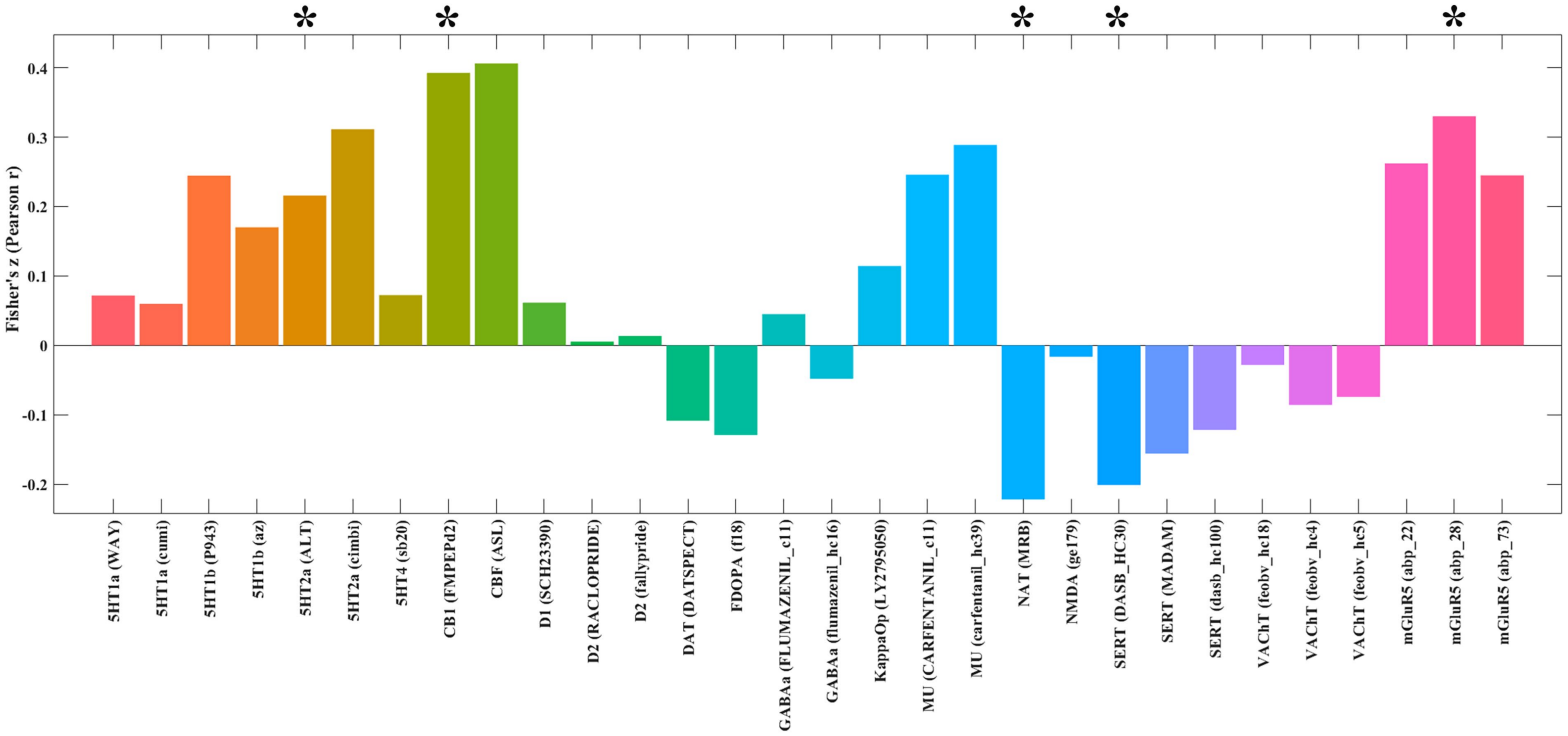
Spatial correlations between the identified extraversion-related networks and neurotransmitter distribution maps. *p < 0.05 after false discovery rate (FDR) correction. 5HT1a, 5-Hydroxytryptamine Receptor 1A; 5HT1b, 5-Hydroxytryptamine Receptor 1B; 5HT2a, 5-Hydroxytryptamine Receptor 2a; 5HT4, 5-Hydroxytryptamine Receptor 4; CB1, Cannabinoid Receptor type 1; D1, Dopamine Receptor D1; D2, Dopamine Receptor D2; DAT, Dopamine Transporter; FDOPA, Fluorodopa; GABAa, Gamma-Aminobutyric Acid type A receptor; KappaOp, Kappa Opioid Receptor; MU, Mu Opioid Receptor; NAT, Noradrenaline Transporter; NMDA, N-Methyl-D-Aspartate Receptor; SERT, Serotonin Transporter; VAChT, Vesicular Acetylcholine Transporter; mGluR5, Metabotropic Glutamate Receptor 5.

Analysis using the JuSpace toolbox characterized the neurochemical profile of the extraversion-associated functional networks (derived from FCNM) through its spatial correlations with neurotransmitter system maps (Figure 3). These networks exhibited significant positive correlations with 5-hydroxytryptamine receptor 2A (5HT2a; *p* = 0.021, *r* = 0.215), cannabinoid receptor type-1 (CB1; *p* = 0.005, *r* = 0.392), and metabotropic glutamate receptor 5 (mGluR5; *p* = 0.01, *r* = 0.330). In contrast, it showed negative correlations with the norepinephrine transporter (NAT; *p* = 0.018, *r* = −0.221) and serotonin transporter (SERT; *p* = 0.023, *r* = −0.201).

## Discussion

This study, employing FCNM on the HCP dataset, offers a fresh perspective on the neural underpinnings of extraversion by pinpointing the network-level localization of GM volume correlates. While prior VBM studies presented a somewhat fragmented picture of regional brain associations with extraversion, our findings reveal a compelling convergence: these seemingly disparate regions coalesce into discernible functional networks. Specifically, our analysis highlights the involvement of core nodes within the DMN (including the mPFC and angular gyrus) and the FPN (encompassing the DLPFC, FIC, and PPC). Complementing this, our neurochemical mapping revealed that these extraversion-associated networks exhibit significant positive spatial correlations with 5HT2a, CB1, and mGluR5 densities, and negative correlations with NAT and SERT densities.

VBM studies investigating the relationship between extraversion and GM volume have been characterized by a notable lack of convergence in regional findings (DeYoung et al., 2010; Li et al., 2019; Lu et al., 2014; Grodin and White, 2015; Kapogiannis et al., 2013; Zou et al., 2018; Andari et al., 2014; Cremers et al., 2011; Forsman et al., 2012; Nostro et al., 2017; Coutinho et al., 2013; Omura et al., 2005). This apparent heterogeneity likely arises from a confluence of factors, ranging from subtle variations in sample demographics (e.g., age, gender, health profiles) and methodological nuances in image analysis protocols (e.g., choices in smoothing kernels, statistical thresholding, and covariate control) (Chen and Canli, 2022), to the inherently complex and distributed nature of personality trait representation in the brain. Indeed, it is increasingly recognized that personality traits, as multifaceted constructs, are unlikely to be neatly confined to circumscribed brain regions (Adelstein et al., 2011; Mulders et al., 2018) and is intrinsically woven into the brain’s functional architecture, implying a network-based organization (Adelstein et al., 2011; Mulders et al., 2018; Li et al., 2022; Toschi et al., 2018; Markett et al., 2018). Our study elegantly demonstrates that by shifting the analytical lens from a region-centric to a network-based perspective, the seemingly scattered GM alterations reported in VBM studies can be meaningfully integrated into a coherent functional network. FCNM emerges as a particularly pertinent methodology to tackle this challenge, adeptly synthesizing coordinate information from diverse studies and harnessing the power of large-scale connectome datasets to delineate common brain networks associated with specific symptoms or traits (Zhang et al., 2024; Yang et al., 2025). The successful deployment of FCNM in the present study to investigate personality traits not only furnishes a compelling strategy for addressing the persistent issue of heterogeneity in personality neuroscience, but also paves the way for a more cohesive and biologically grounded understanding of extraversion’s neural substrates.

Our study, employing FCNM on the large-scale HCP dataset, provides evidence that GM volume correlates of extraversion are localized within the DMN. Notably, the DMN is anchored by core nodes such as the mPFC identified in our study, regions that are critically implicated in self-referential processing, social cognition, and the integration of information across cognitive domains (Menon, 2023; Smallwood et al., 2021), which are highly relevant to the behavioral characteristics of extraversion (Lei et al., 2015). Similarly, the angular gyrus is known for its role in perspective taking and integrating multisensory information, further supporting its relevance to extraversion’s social and attentional dimensions (Seghier, 2013). This network-level mapping significantly refines previous VBM findings, which have yielded inconsistent results regarding the structural basis of extraversion. Our results converge with a growing body of literature highlighting the DMN’s central role in the neurobiology of extraversion (Lei et al., 2015; Lei et al., 2013). Specifically, while our study focuses on GM volume, it aligns with functional neuroimaging studies demonstrating altered DMN activity and connectivity patterns associated with extraversion (Lei et al., 2013; Knyazev, 2013; Sampaio et al., 2014; Wei et al., 2011; Pang et al., 2017; Hsu et al., 2018; Yoon et al., 2022), where the functional dynamics within and between nodes like the mPFC and angular gyrus likely contribute significantly to these observed associations. Importantly, the predictive utility of DMN FC for extraversion has been demonstrated, suggesting that resting-state DMN activity patterns can serve as a neural signature for this personality trait and potentially predict extraversion levels in novel individuals (Hsu et al., 2018). Our finding that GM correlates of extraversion are network-localized within the DMN suggests a potential structural foundation for these observed functional differences and predictive capabilities. Crucially, the influence of extraversion extends beyond the DMN in isolation, encompassing its interactions with other large-scale brain networks (Lei et al., 2015). For example, Pang et al. demonstrated that extraversion modulates the FC hubs of resting-state networks, implying a broader network-level reorganization that includes the DMN and its interplay with systems involved in cognitive control and emotional processing (Pang et al., 2017). Furthermore, Rogala et al. (2021) highlighted that stronger connectivity, potentially involving DMN interactions with other networks supporting cognitive resilience, and higher extraversion together protect against stress-related cognitive decline. This suggests that the functional profile of extraversion is not solely determined by DMN properties alone, but also by how the DMN integrates and interacts with networks responsible for executive functions, emotion regulation, and stress response. Understanding these inter-network dynamics is essential for a comprehensive account of the neural mechanisms underlying extraversion.

Expanding beyond the DMN, our findings robustly implicate the FPN as a critical functional substrate for extraversion. This network, essential for higher-order cognitive functions such as cognitive control, executive functions, and goal-directed behavior (Dixon et al., 2018; Zanto and Gazzaley, 2013; Marek and Dosenbach, 2018), emerges as a functionally convergent hub for the structural correlates of extraversion (Toschi et al., 2018; Pang et al., 2017). Importantly, our findings align with and extend existing evidence suggesting the FPN’s predictive power in relation to extraversion (Hsu et al., 2018). This predictive ability is likely intrinsically linked to the FPN’s core functions in executive control and cognitive flexibility, underscoring the FPN’s fundamental role in shaping, not just reflecting, extraversion (Pang et al., 2017; Hsu et al., 2018). The FPN does not operate in isolation; its FC and interactions with other brain networks, like the DMN, are crucial for understanding its role in extraversion (Toschi et al., 2018; Lei et al., 2013; Pang et al., 2017). The FPN, acting as a flexible hub (Toschi et al., 2018), may orchestrate these interactions, balancing internal and external focus, reward processing, and attentional allocation in the context of extraverted behaviors.

Examining the specific component regions of the FPN further elucidates its relevance to extraversion. The DLPFC, a core node within the FPN, is indirectly supported by our findings, consistent with previous research (Pang et al., 2017; Kong et al., 2015; Lee et al., 2024). While our study focuses on the functional network mapping of GM alterations, prior work has directly linked DLPFC structure to extraversion (Kong et al., 2015). Our results suggest that heterogeneous GM changes, wherever they occur, functionally impact the FPN, which prominently connects the DLPFC. The FIC and PPC, while less explored in direct GM-extraversion links in the previous literature, are crucial FPN nodes for salience processing, social cognition, and interoception (Lai et al., 2019; Lee et al., 2024; Cho, 2021; Tian et al., 2025). Their integral role within the FPN suggests that the network’s functional organization may also influence the social and emotional facets of extraversion, extending beyond purely cognitive control aspects.

To rigorously validate the robustness of our findings derived from the HCP dataset, we undertook replication analyses utilizing an independent dataset sourced from Southwest University (SALD). Encouragingly, the results from this independent replication cohort mirrored our primary findings: the functional networks identified in the SALD also exhibited a primary localization within the DMN and FPN, demonstrating a notable consistency with the patterns observed in the HCP dataset. This cross-dataset convergence strongly suggests that the extraversion-related functional networks we have delineated possesses robust cross-dataset generalizability, thereby mitigating potential concerns regarding dataset-specific biases inherent to single-dataset studies. Moreover, to further probe the stability of our results and assess the influence of analytical parameters, we systematically varied the sphere radius size (exploring 1 mm, 4 mm, and 7 mm radii) in the FCNM analysis. Reassuringly, the resultant functional networks remained remarkably similar across these varying radii, further bolstering the reliability and spatial specificity of our findings. This parameter sensitivity analysis robustly demonstrates that our results are not unduly contingent upon specific parameter choices within the FCNM approach. Collectively, these validation analyses significantly enhance the generalizability and overall credibility of our research, substantially strengthening confidence in our network-level characterization of the structural correlates of extraversion.

Beyond network localization, our Juspace analysis explored spatial relationships between extraversion-linked networks and neurotransmitter receptor distributions, revealing specific neurochemical signatures. Notably, these networks exhibited significant positive spatial correlations with the density of 5HT2a, mGluR5, and particularly strongly with CB1. This suggests that brain networks functionally integral to extraversion are characterized by a higher abundance of these receptors. Higher 5HT2a density could facilitate serotonergic modulation of cognition (Gong et al., 2014) and social processing, given its dense cortical expression (Erritzoe et al., 2009); increased mGluR5 might support enhanced synaptic plasticity (Niswender and Conn, 2010) and excitatory signaling crucial for cognitive flexibility and emotional regulation (Terbeck et al., 2015); and greater CB1 density likely enhances endocannabinoid system sensitivity, promoting approach motivation, reward responsiveness, and sociability (Laricchiuta and Petrosini, 2014; Navarro et al., 2022). Collectively, these findings point towards an augmented signaling capacity through these receptor systems in the DMN and FPN regions, underpinning key facets of extraverted behavior. Conversely, the extraversion-associated networks showed significant negative correlations with the NAT and the SERT. This pattern implies lower transporter density in these functional networks, which would translate to reduced reuptake and thus increased synaptic availability and prolonged signaling of norepinephrine and serotonin, respectively (Shao and Zhu, 2020). Sustained noradrenergic signaling could underpin the heightened arousal, alertness, and energetic engagement characteristic of extraversion (DeYoung et al., 2010), while enhanced serotonergic tone might contribute to positive affect (Canli, 2004) and social affiliation. While the broader literature on personality genetics and neurotransmitter systems presents a complex and sometimes inconsistent picture (Gillihan et al., 2007; Balestri et al., 2014), our findings of spatially co-localized neurochemical patterns offer a systems-level perspective. Thus, the observed constellation—enhanced receptivity via 5HT2a, CB1, and mGluR5, alongside increased synaptic availability of NAT and SERT—suggests a distinct neurochemical architecture within the DMN and FPN regions that collectively supports the multifaceted expression of extraversion.

### Limitations

While this study provides valuable insights into the neural underpinnings of extraversion, it is important to acknowledge several inherent limitations that also highlight promising avenues for future research. Firstly, analysis herein relied primarily on VBM results reported in the included studies, thus focusing exclusively on GM volume as a singular structural measure. This inherently limits the comprehensiveness of our understanding of the neurobiology associated with extraversion. Future investigations could significantly broaden the scope by incorporating multi-modal neuroimaging approaches to encompass a wider array of brain structural and functional properties. Such a multi-modal strategy would provide a richer and more multi-faceted characterization of the neurobiological architecture associated with extraversion. Secondly, it is crucial to recognize that FCNM, by its nature, is a correlational methodology and, as such, cannot definitively establish causal relationships between observed GM alterations and functional impairments. To move beyond correlational inferences, future studies could fruitfully employ causal inference techniques, such as lesion network mapping or dynamic causal modeling. Thirdly, the present study primarily focused on extraversion within healthy populations, limiting the direct clinical applicability of the findings. To extend the clinical relevance, future research should investigate clinical populations, such as individuals diagnosed with social anxiety disorder, major depressive disorder, or autism spectrum disorder. Exploring how the brain network correlates of extraversion manifest in diverse mental health contexts could refine our understanding of the role personality traits play in the vulnerability, clinical presentation, and longitudinal course of various psychiatric disorders. Comparative analyses examining the differences and overlaps in extraversion-related network alterations between clinical and non-clinical populations could yield valuable insights into the intricate interplay between personality and psychopathology. Finally, several methodological considerations regarding the FCNM analysis warrant attention. The accuracy of our FCNM results is inherently dependent on the quality and reliability of the GM findings reported in the primary studies included. Furthermore, the current analysis did not incorporate negative findings from the original studies, nor did it weight studies based on sample size. The potential influence of unaccounted confounding factors (e.g., demographic variations, differences in scanning parameters and analytical process across studies) also cannot be fully discounted. Addressing these issues suggests that future research, particularly when using functional data for similar network mapping, should ideally utilize datasets from samples that are closely matched in demographic and clinical profiles to the participants of the original studies. Continued analytical advancements will also be crucial for better addressing issues like the integration of negative results, sample size weighting, and confound control (Zhang et al., 2024).

## Conclusion

In conclusion, this study employed FCNM on the large-scale HCP dataset, complemented by independent replication and sensitivity analyses, to offer a novel network-level perspective on the structural neural correlates of extraversion. By demonstrating that previously disparate GM volume findings converge upon the DMN and FPN, our research provides a more coherent neurobiological framework for understanding this fundamental personality trait. This network localization approach not only helps reconcile inconsistencies in prior VBM literature but also underscores the importance of examining personality through the lens of distributed brain systems rather than isolated regions. Our additional analyses suggest that these networks are associated with a distinct neurochemical architecture, characterized by higher densities of 5HT2a, CB1, and mGluR5 receptors and lower densities of NAT and SERT transporters. This work lays a solid foundation for future multi-modal, causal-inference, and clinically focused investigations aimed at further elucidating the complex interplay between brain structure, function, neurochemistry, and personality. Ultimately, a network-level understanding of personality, as exemplified by this study of extraversion, holds immense promise for advancing our knowledge of the neural mechanisms underlying human behavior and individual differences in both health and disease.

## Data Availability

The raw data supporting the conclusions of this article will be made available by the authors, without undue reservation.

## Footnotes

^1^ https://www.fil.ion.ucl.ac.uk/spm/

^2^ https://rfmri.org/DPABI

^3^ https://github.com/juryxy/JuSpace

## Glossary

16PF: Sixteen Personality Factor Questionnaire
5HT1a: 5-Hydroxytryptamine Receptor 1A
5HT1b: 5-Hydroxytryptamine Receptor 1B
5HT2a: 5-hydroxytryptamine receptor 2A
5HT4: 5-Hydroxytryptamine Receptor 4
CB1: cannabinoid receptor type-1
corr: correction
D1: Dopamine Receptor D1
D2: Dopamine Receptor D2
DAT: Dopamine Transporter
DLPFC: dorsolateral prefrontal cortex
DMN: default mode network
DPABI: data processing & analysis for brain Imaging
EPQ-RSC: Eysenck Personality Questionnaire-Revised Short Scale for Chinese
F: female
FA: flip angle
FC: functional connectivity
FCNM: functional connectivity network mapping
FD: framewise displacement
FDOPA: Fluorodopa
FDR: false discovery rate
FIC: frontoinsular cortex
fMRI: functional magnetic resonance imaging
FOV: field of view
FPN: frontoparietal network
FEW: family-wise error
FWHM: full width at half maximum
GABAa: Gamma-Aminobutyric Acid type A receptor
GLM: general linear model
GM: gray matter
GRF: gaussian random field
GRE-EPI: gradient-recalled echo-Planar Imaging
HCP: Human Connectome Project
KappaOp: Kappa Opioid Receptor
L: left
M: male
mGluR5: metabotropic glutamate receptor 5
MNI: Montreal Neurological Institute
MPQ-BF: Multidimensional Personality Questionnaire Brief Formm
PFC: medial prefrontal cortex
MU: Mu Opioid Receptor
NAT: norepinephrine transporter
NEO-FFI: NEO Five Factor Inventory
NEO-PI-R: Revised NEO Personality Inventory
NMDA: N-Methyl-D-Aspartate Receptor
OFC: orbitofrontal cortex
PET: Positron Emission Tomography
PPC: posterior parietal cortex
R: right
ROI: region of interest
rs-fMRI: resting-state functional MRI
SALD: Southwest University Adult Lifespan Dataset
SD: standard deviation
SERT: serotonin transporter
SPM12: Statistical Parametric Mapping
TE: echo time
TGMV: total gray matter volume
TIV: total intracranial volumes
TR: repetition time
uncorr: uncorrection
VAChT: vesicular acetylcholine transporter
VBM: voxel-based morphometry
