# Supplementary material for: Network-based mapping and neurotransmitter architecture of brain gray matter correlates of extraversion
Source: Front Syst Neurosci. 2025 Oct 3;19:1640639. doi: 10.3389/fnsys.2025.1640639 (PMC12531143; doi:10.3389/fnsys.2025.1640639)
Supplement: Supplementary file 1 [file Table_1.DOCX]

**Table 1** Sample and imaging characteristics of the studies included extraversion analysis.

| **Included study** | **Sample size** | **Ratio F/M** | **Mean age (SD)** | **Scales** | **Scanner/FWHM (mm)** | **Nuisance covariate** | **Statistical analysis/p-value corr** |
| --- | --- | --- | --- | --- | --- | --- | --- |
| Andar et al., 2014 | 30 | 13/17 | 23.57 (3.70) | NEO-PI-R | 1.5 T/8 | Gender, age, TGMV | p < 0.05, nonstationary corr. |
| Coutinho et al.,2013 | 52 | 29/23 | 25.0 (5.1) | NEO-FFI | 1.5 T/10 | Gender, age, TIV | GLM/p < 0.05, Monte Carlo  corr |
| Cremers et al., 2011 | 65 | 42/23 | 40.5 (9.7) | NEO-FFI | 3.0 T/8 | Age, scan center, TGMV | p < 0.05, FWE corr and  p < 0.001, uncorr |
| DeYoung et al., 2010 | 116 | 58/58 | 22.9 (5.5) | NEO-PI-R | 3.0 T/8 | Gender, age, TGMV | GLM/p < 0.05, cluster-size corr (Monte Carlo simulation) |
| Forsman et al.2012 | 32 | 0/32 | 33.2 (7.8) | 16PF | 1.5 T /12 | Age | GLM/p < 0.05, FDR, corr |
| Grodin et al., 2015 | 83 | 46/37 | 24.9 (7.7) | MPQ-BF | 3.0 T/12 | Gender, age, alternate  extraversion traits | p < 0.05, FDR,  corr&p < 0.001, uncorr |
| Kapogiannis, et al.2013 | 87 | 42/45 | 72 (7.7) | NEO-PI-R | 1.5 T /12 | Gender, age, TIV,  years of education | GLM/p < 0.05, FWE corr |
| Li et al., 2019 | 337 | 189/148 | 20.0 (1.3) | NEO-PI-R | 3.0 T/10 | Gender, age, other four traits, TGMV | p < 0.05, FWE corr |
| Lu et al., 2014 | 71 | 37/34 | 22.35 (1.5) | EPQ-RSC | 3.0 T/8 | Gender, age, TIV | GLM/p < 0.05, AlphaSim corr |
| Omura et al., 2005 | 41 | 22/19 | 23.8 (5.4) | NEO-PI-R | 3.0 T/12 | Gender, age | GLM/p < 0.001, uncorr |
| Nostro et al., 2017 | 364 | 182/182 | 29.1 (3.45) | NEO-FFI | 3.0 T/8 | Gender, age, TIV | GLM/p < 0.05, FWE corr |
| Su et al., 2018 | 100 | 58/42 | 22.0 (2.33) | EPQ-RSC | 3.0 T/- | Gender, age, TIV | GLM/p < 0.05, FDR corr |
| Zou, et al., 2018 | 100 | 50/50 | 21.91(2.29) | EPQ-RSC | 3.0T/6 | Gender, age | GLM/p < 0.05, FWE corr |

Corr,correction; 16PF, Sixteen Personality Factor Questionnaire; corr, correction; EPQ-RSC, Eysenck Personality Questionnaire-Revised Short Scale for Chinese; F, female; FDR, false discovery rate; FWE, family-wise error; FWHM, full width at half maximum; GLM, general linear model; GRF, Gaussian random field; M, male; MPQ-BF, Multidimensional Personality Questionnaire Brief Form; NEO-FFI, NEO Five Factor Inventory; NEO-PI-R, Revised NEO Personality Inventory; TGMV, total gray matter volume; TIV, total intracranial volumes; uncorr, uncorrection.
