# Supplementary material for: Network-based mapping and neurotransmitter architecture of brain gray matter correlates of extraversion
Source: Front Syst Neurosci. 2025 Oct 3;19:1640639. doi: 10.3389/fnsys.2025.1640639 (PMC12531143; doi:10.3389/fnsys.2025.1640639)
Supplement: Supplementary file 2 [file Table_2.DOC]

**Table 1.** Demographic information of the discovery and validation datasets

| **Dataset sample size** | **Age (years)** | **Gender (F/M)** |
| --- | --- | --- |
| HCP 1093 | 28.78±3.69 | 594/499 |
| SALD 329 | 37.81±13.79 | 207/122 |

Age is expressed as mean ± standard deviation. Note: HCP, Human Connectome Project; SALD,

Southwest University Adult Lifespan Dataset. F, female; M, male.
