# Supplementary material for: Network-based mapping and neurotransmitter architecture of brain gray matter correlates of extraversion
Source: Front Syst Neurosci. 2025 Oct 3;19:1640639. doi: 10.3389/fnsys.2025.1640639 (PMC12531143; doi:10.3389/fnsys.2025.1640639)
Supplement: Supplementary file 3 [file Table_3.DOCX]

**Table 2.** Resting-state fMRI parameters of discovery and validation datasets

| **Parameter** | **HCP** | **SALD** |
| --- | --- | --- |
|  |  |  |
| Scanner | 3.0T Siemens Trio | 3.0T Siemens Trio |
| Sequence | GRE-EPI | GRE-EPI |
| TR (ms) | 720 | 2000 |
| TE (ms) | 33.1 | 30 |
| FA (°) | 52 | 90 |
| FOV (mm²) | 208×180 | 220×220 |
| Matrix size | 104×90 | 64×64 |
| Slice thickness (mm) | 2 | 3 |
| Slice gap (mm) | 0 | 1 |
| Slices | 72 | 32 |
| Time points | 1210 | 242 |

FA, flip angle; fMRI, functional magnetic resonance imaging; FOV, field of view; GRE-EPI, gradient-recalled echo-Planar Imaging; HCP, Human Connectome Project; SALD, Southwest University Adult Lifespan Dataset; TE, echo time; TR, repetition time.
