# Supplementary figures and images for: Network-based mapping and neurotransmitter architecture of brain gray matter correlates of extraversion
Source: Front Syst Neurosci. 2025 Oct 3;19:1640639. doi: 10.3389/fnsys.2025.1640639 (PMC12531143; doi:10.3389/fnsys.2025.1640639)

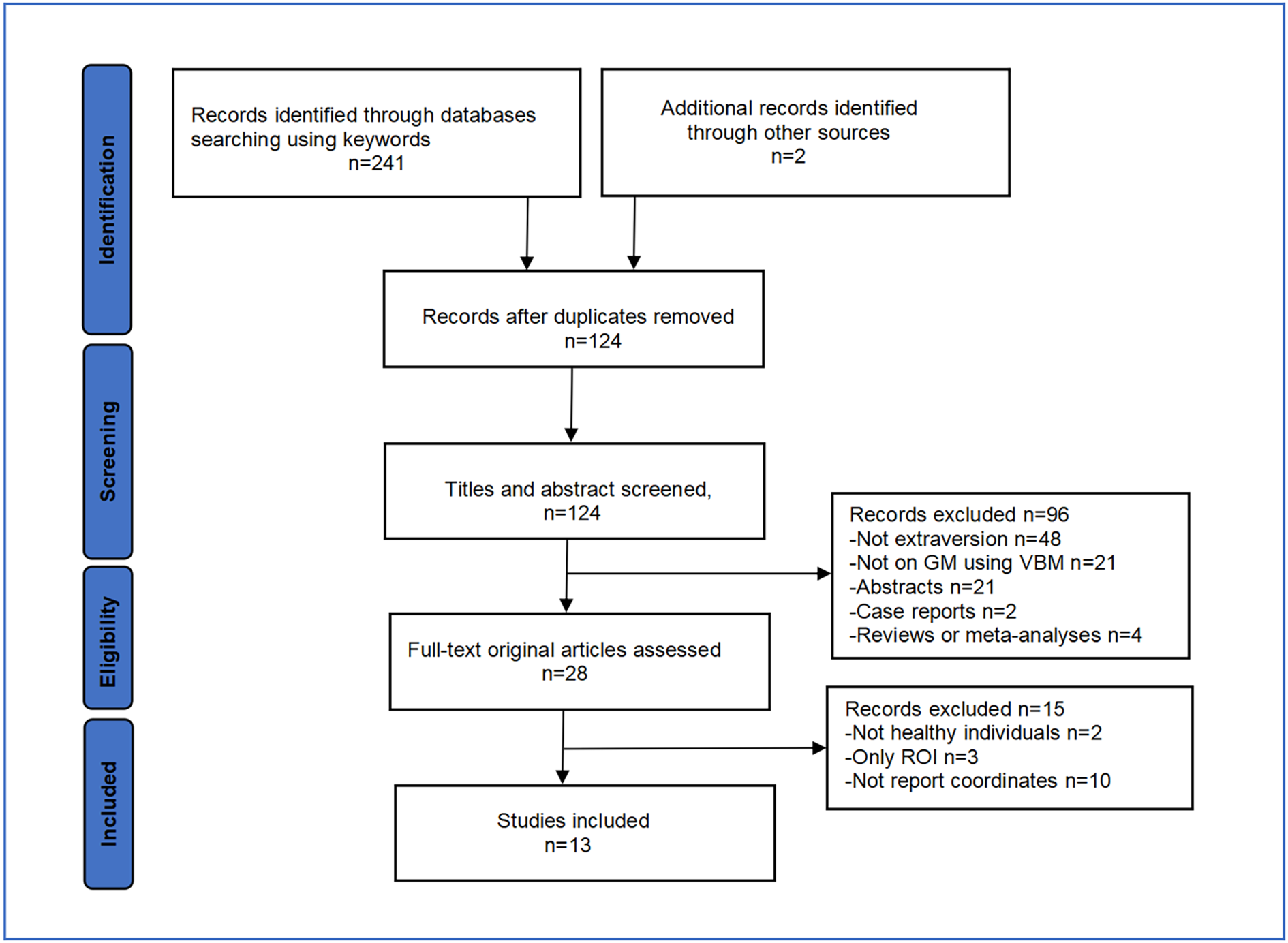

Supplement: Supplementary file 4 [file Image_1.TIF]

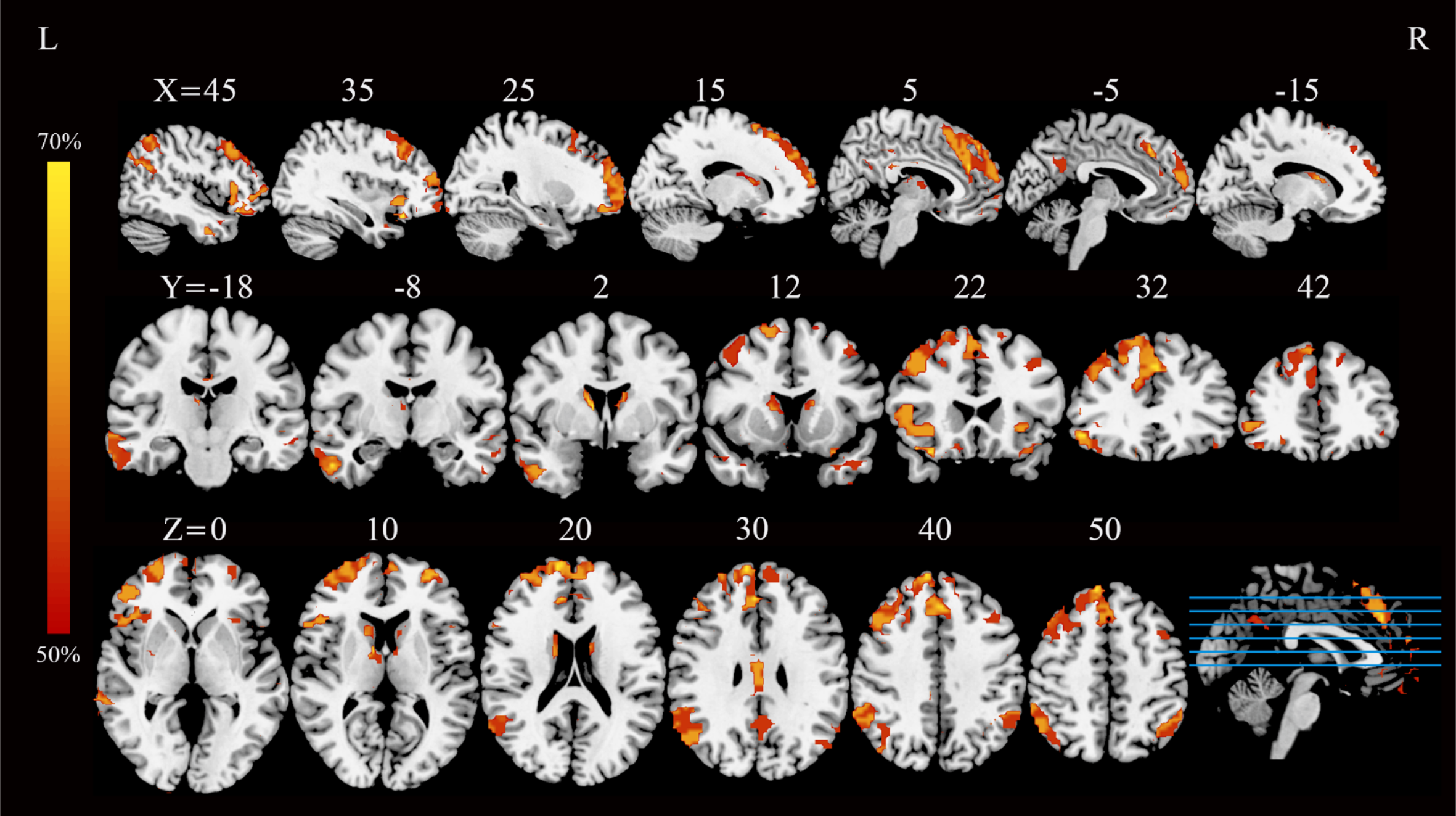

Supplement: Supplementary file 5 [file Image_2.TIF]

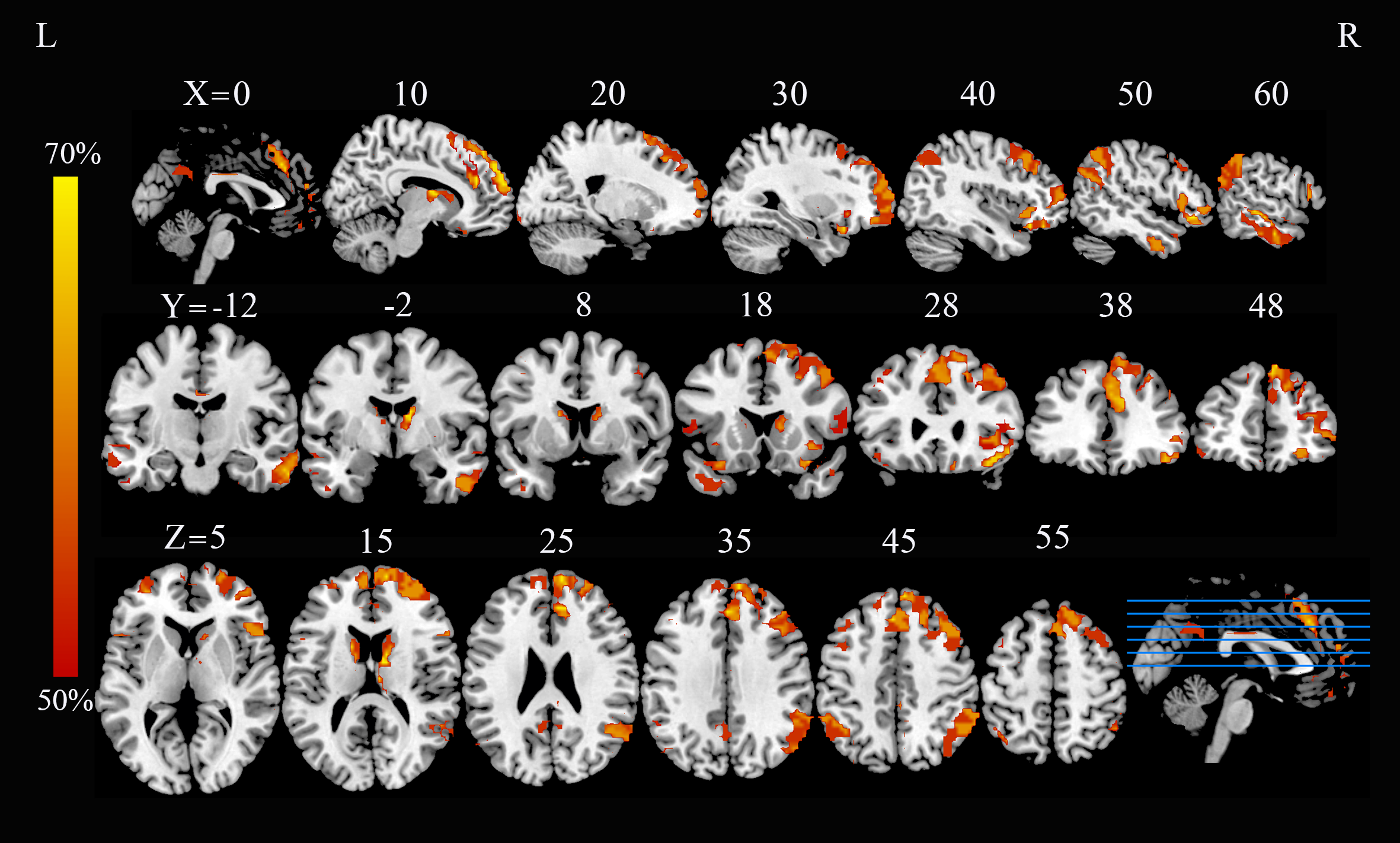

Supplement: Supplementary file 6 [file Image_3.TIF]

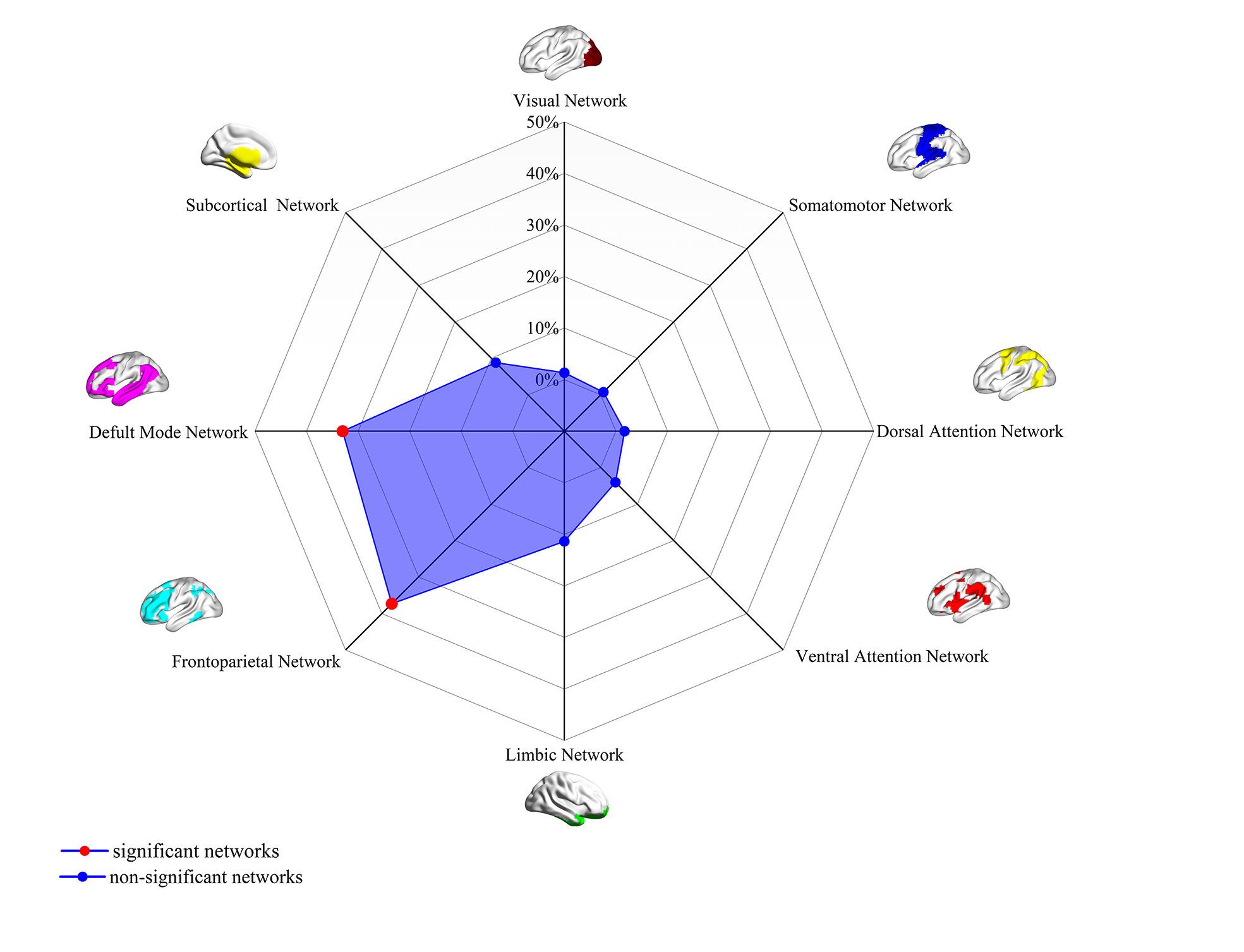

Supplement: Supplementary file 7 [file Image_4.TIF]

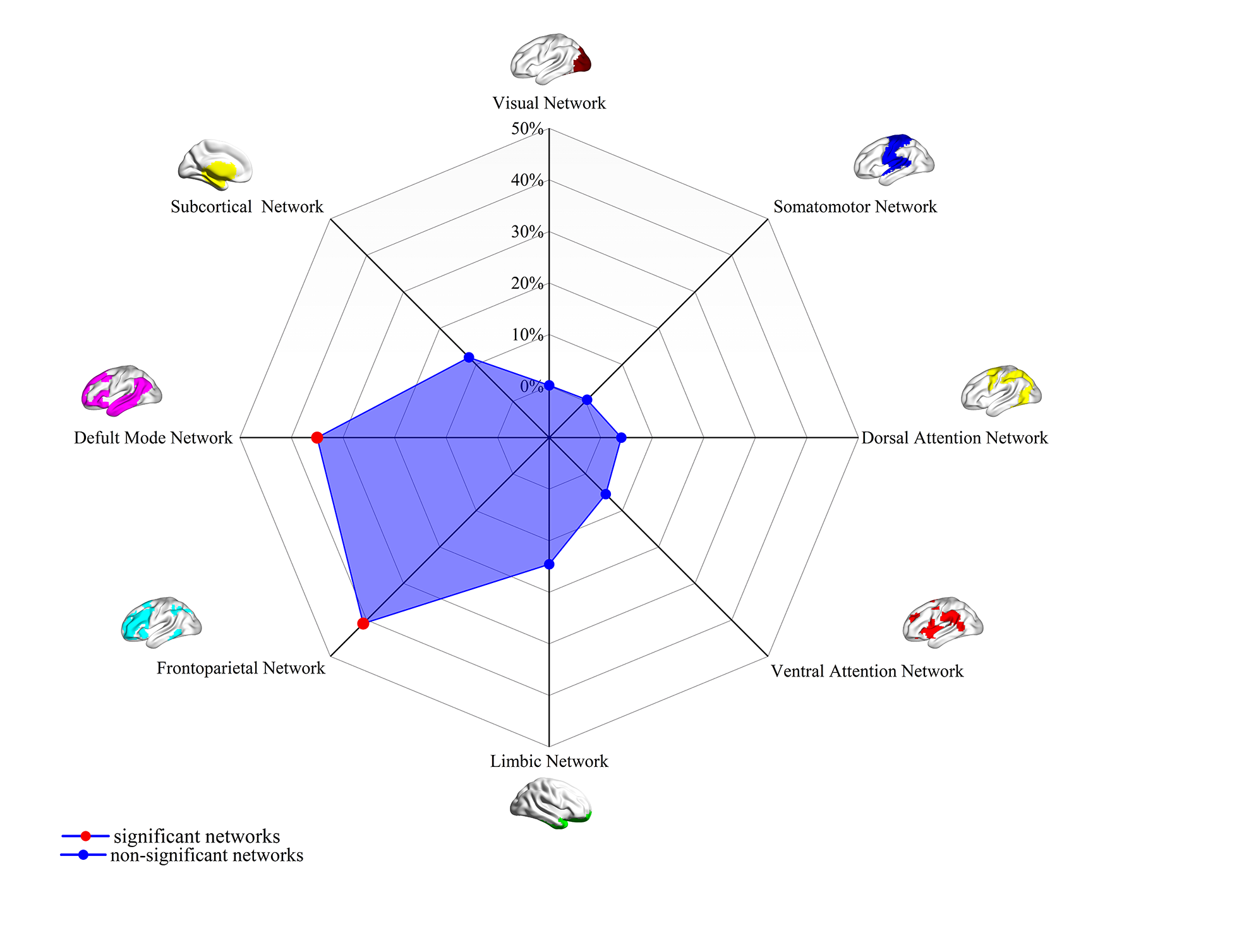

Supplement: Supplementary file 8 [file Image_5.TIF]

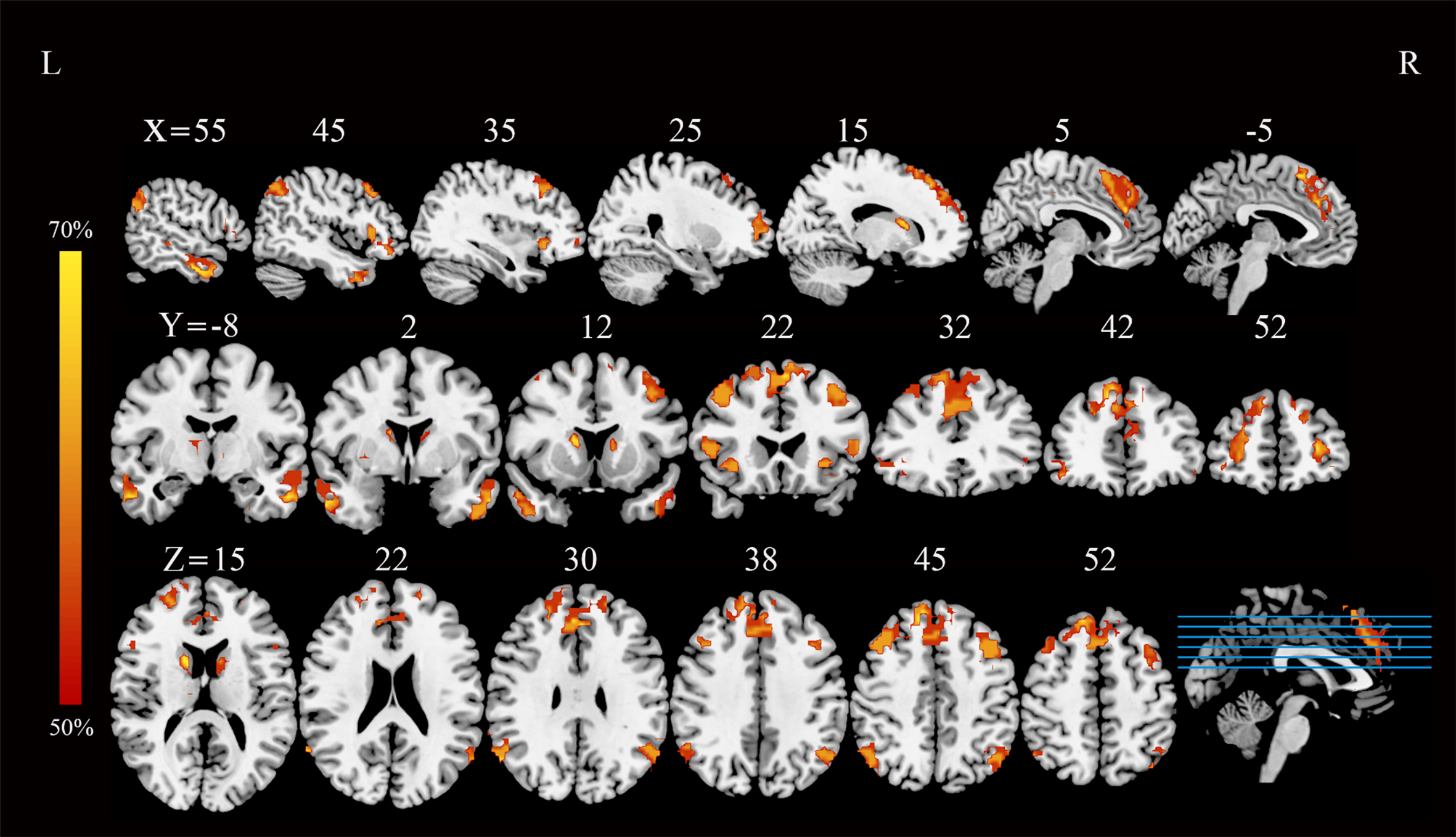

Supplement: Supplementary file 9 [file Image_6.TIF]

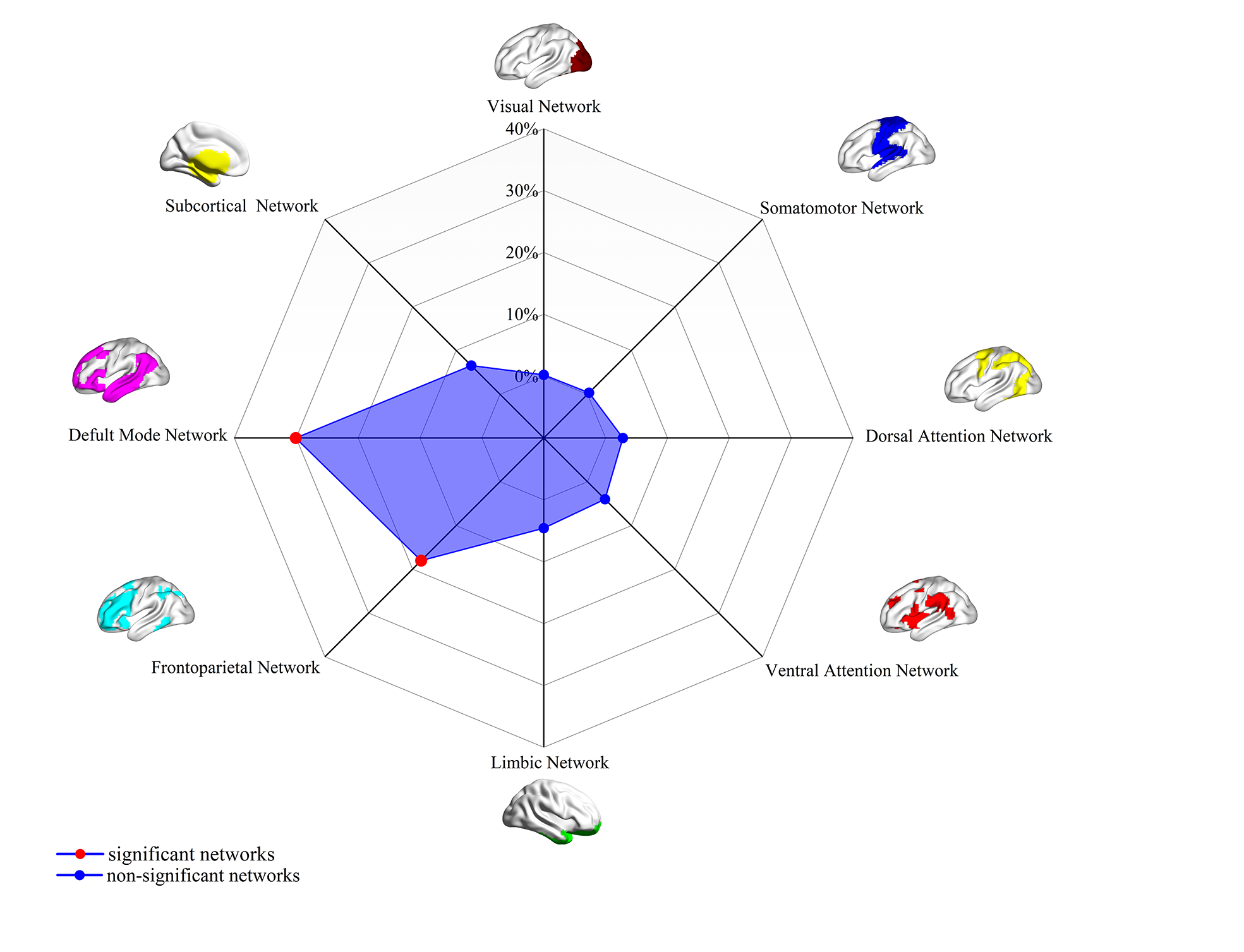

Supplement: Supplementary file 10 [file Image_7.TIF]
